# Genome-Wide Identification of SSR and InDel Markers and Experimental Validation of SSR Markers for Distinguishing Cold-Tolerant and Cold-Sensitive Lily Cultivars

**DOI:** 10.3390/biology15171449

**Published:** 2026-08-24

**Authors:** Yanan Lu, Mingliang He, Lei Wang, Meng Zhao, Qianting Liu, Hongmei Bai, Yan Liu, Jing Gao, Li Zhang, Zhaoxin Wu, Zhi Liu, Yanan Wang

**Affiliations:** 1 Inner Mongolia Academy of Agricultural and Animal Husbandry Sciences, Hohhot 010031, China; ynlunefu@163.com (Y.L.); 15663648869@163.com (M.Z.); lqt18468167060@163.com (Q.L.); 15848909291@163.com (H.B.); liuyannky@163.com (Y.L.); gaojing1204@163.com (J.G.); zl236426@163.com (L.Z.); wuzhaoxin1994@163.com (Z.W.); 2 Institute of Microbiology, Xinjiang Academy of Agricultural Sciences, Urumqi 830091, China; 15739514166@163.com (M.H.); xjauwl@163.com (L.W.); 3 Guangdong Provincial People’s Hospital, Guangdong Academy of Medical Sciences, Guangzhou 510080, China

**Keywords:** cold resistant lilies, simple sequence repeat, insertion, deletion (InDel)

## Abstract

In this study, whole-genome resequencing was performed on the cold-tolerant variety ND-6 and the cold-sensitive variety ‘Sorbonne’. After evaluation, the *Lilium davidii* var. *unicolor* reference genome was selected to analyze SSR distribution characteristics. Whole-genome InDel identification and comparative analysis were conducted for the two varieties, yielding 34,812,909 and 24,497,857 InDels, respectively. Short InDels were predominant, with deletions slightly outnumbering insertions, mostly located in intergenic regions. Twenty pairs of SSR primers were screened and synthesized. Among them, 10 pairs amplified clearly, with a polymorphism rate of 82.6%, effectively distinguishing the two cultivars examined in this study. This study provides systematic data and a reliable marker resource for the analysis of lily genomic variation, laying a foundation for the identification of cold-tolerant germplasm; validation across additional cultivars and individuals will be required to extend their utility to broader germplasm.

## Simple Summary

We resequenced two lily cultivars with contrasting cold tolerance: ND-6 (cold-tolerant) and ‘Sorbonne’ (cold-sensitive). By selecting the most suitable reference genome, we characterized genome-wide SSR distributions and identified millions of InDels between them. Ten polymorphic SSR markers were validated, achieving an 82.6% polymorphism rate and reliably distinguishing the two cultivars examined in this study. This study provides the first systematic InDel analysis in lily and a valuable molecular foundation. The utility of these markers for broader cold-tolerant/cold-sensitive germplasm discrimination requires validation across additional cultivars and individuals.

## 1. Introduction

Lilies *(Lilium* spp.) are perennial bulbous plants of the Liliaceae family that possess ornamental, edible, and medicinal value and are widely cultivated in temperate and subtropical regions worldwide. Among the many breeding objectives, cold hardiness is of both economic and ecological importance. Low-temperature stress during the overwintering period and early spring growth stages significantly limits the geographical distribution and range of application of lily varieties [1]. The Oriental lily hybrid ‘Sorbonne’ has become one of the most popular varieties in the cut flower market due to its elegant flower form and pleasant fragrance; however, its sensitivity to low temperatures limits its cultivation range. In contrast, the local Chinese variety ‘ND-6’ from Inner Mongolia shows strong overwintering ability under open-field conditions, making it a valuable genetic resource for elucidating the genetic architecture of cold tolerance and breeding cold-tolerant varieties. Therefore, clarifying the genomic differences between cold-tolerant and cold-sensitive lily varieties is a molecular prerequisite for improving this trait.

In lilies, cold/freezing tolerance is a complex polygenic trait. Transcriptome analyses have revealed that the cold response in Lilium is primarily mediated by the ICE–CBF/DREB–COR signaling cascade, together with Ca^2+^- and ABA-dependent signal transduction [2]. In the cold-hardy species *L. lancifolium*, candidate cold-resistance genes include transcription factors (LlICE, LlDREB1/CBF, LlNAC, LlMYB), cryoprotective proteins (LEA, COR), and enzymes involved in membrane lipid desaturation (LlFAD3), proline synthesis (LlP5CS), and soluble-sugar metabolism [2]. In *L. davidii* var. *unicolor* (Lanzhou lily), key anti-freeze genes include members of the CBF family, the *OLEO3* gene, and C2H2-type zinc-finger transcription factors [1]. Physiological indicators associated with lily cold tolerance include membrane stability (relative electrolyte leakage), malondialdehyde (MDA) content, proline and soluble-sugar accumulation, and antioxidant enzyme activities [1]. To date, no QTL mapping study for cold hardiness has yet been reported in *Lilium*, largely owing to its enormous and highly repetitive genome; nevertheless, the candidate genes described above provide a foundation for marker-assisted and genomics-assisted breeding for cold tolerance.

Although lilies are economically important, their large and complex genomes have hindered genomic research. Lily genomes range in size from 30 to 48 Gb, approximately 10 to 16 times the size of the human genome. Due to high heterozygosity, abundant repetitive sequences, and polyploidization events, genome assembly has remained extremely challenging [3,4,5]. This complexity has hindered the assembly of reference genomes and the systematic development of genome-wide molecular markers. In recent years, the release of chromosome-level reference genomes for *Lilium davidii* var. *unicolor* (36.68 Gb), *Lilium regale* (35.6 Gb), and *Lilium sargentiae* (35.66 Gb) has marked the entry of lily molecular biology into the post-genomic era [4,5,6]. The publication of these achievements has laid the foundation for integrated whole-genome resequencing and high-throughput variant detection in lilies.

Molecular markers are essential tools for germplasm identification, genetic diversity assessment, and marker-assisted selection (MAS) [7,8]. Simple sequence repeats (SSRs) and insertion/deletion (InDel) polymorphisms offer numerous advantages, including codominant inheritance, high polymorphism, high genome-wide abundance, and ease of routine genotyping [9,10]. In lilies, SSR markers have primarily been developed from expressed sequence tags (ESTs). In the ‘Elodie’ lily transcriptomic dataset, EST-SSR markers were developed and validated across 65 lily genotypes, demonstrating high polymorphism [11]. In addition, polymorphic SSR and InDel loci have also been characterized from the complete chloroplast genomes of *Lilium* species [12]. However, the vast majority of existing lily SSR markers are derived from transcriptomic rather than genomic sequences. Consequently, they cannot represent the full spectrum of genomic variation, with significant gaps particularly in non-coding regions and intergenic regions. Furthermore, to the best of our knowledge, no SSR markers specifically targeting cold-tolerant and cold-sensitive lily varieties have yet been developed or validated.

Single-nucleotide polymorphisms (SNPs) are the most abundant and widely used molecular markers in crop genetics and breeding. SNP markers have been extensively applied in genetic diversity assessment, linkage map construction, QTL mapping, marker-assisted selection (MAS), and genome-wide association studies across numerous crop species [8]. In horticultural and ornamental crops, high-throughput genotyping platforms such as genotyping-by-sequencing (GBS) have enabled the genome-wide development of SNP markers and their application in DNA fingerprinting, germplasm characterization, and marker-assisted breeding [13]. These advances highlight the central role of sequence-based markers (SNPs and InDels) in modern molecular breeding.

Compared with SSRs, InDel markers have been widely used in crops such as rice [14], barley [15], cucumber [16], Chinese cabbage [17], and rapeseed [18], but they have received little attention in lilies. InDels are length polymorphisms caused by short-fragment insertions or deletions in the genome. Because they combine the advantages of both SSRs and SNPs, they are ideal markers for constructing low-cost genotyping platforms [9,16]. InDels are frequently located in regulatory regions such as promoters and introns, and can affect gene function by altering cis-regulatory elements, affecting alternative splicing, or causing frameshift mutations [9]. They are therefore an important class of candidate variants for dissecting regulatory variation in cold-tolerance genes and are well suited for constructing a molecular-marker system for cold-tolerance research. However, due to the long-standing lack of high-quality reference genomes, the detection of genome-wide InDel variations and marker development in lilies has lagged behind.

This study used two lily varieties, “ND-6” and ‘Sorbonne’, which exhibit significant differences in cold tolerance, as experimental materials. Through whole-genome resequencing, the study systematically analyzed the SSR and InDel variations between the two. Specific research activities included: evaluating the suitability of three published lily reference genomes as alignment references for resequencing; conducting a comprehensive survey of SSR distribution patterns in the lily genome; identifying, annotating, and comparing genome-wide InDel polymorphisms between the two varieties; and screening, designing, and experimentally validating polymorphic SSR markers capable of effectively distinguishing the two varieties. This study conducted a systematic identification and comparative analysis of genome-wide InDels in lilies and reported SSR markers for the identification of cold-tolerant and cold-sensitive lily germplasm. The genomic resources and molecular markers obtained are expected to facilitate future analyses of lily genetic diversity and variety identification, and to provide a molecular foundation for evaluating their potential utility in marker-assisted selection for cold tolerance. Because only one individual of each cultivar was resequenced, the variant differences reported here should be regarded as differences between the two specific accessions examined, and their generalization to cold-tolerant versus cold-sensitive lily germplasm at large remains a hypothesis requiring validation across additional cultivars and individuals.

## 2. Materials and Methods

### 2.1. Plant Materials

This study conducted winter survival trials at the Flower Cultivation Resource Garden of the Inner Mongolia Academy of Agricultural and Animal Husbandry Sciences (Hohhot, Inner Mongolia, China) from November 2024 to May 2025. A total of five lily cultivars/varieties were initially screened, with 10 bulbs per cultivar and three replicates, and the winter survival rate was calculated as the number of bulbs that survived (regenerated in spring) divided by the number of bulbs planted in autumn × 100%. Based on the winter survival rates, the cold-tolerant variety ‘ND-6’ (100% survival) and the non-cold-tolerant variety ‘Sorbonne’ (0% survival) were selected for subsequent analysis. Leaf samples from both varieties were collected for DNA resequencing. From December 2024 to February 2025, the average winter temperature in the region ranged from −10.7 °C to −6.4 °C.

### 2.2. Genomic DNA Extraction and Library Construction

Genomic DNA was extracted from a single individual of each cultivar (ND-6 and ‘Sorbonne’) and subjected to whole-genome resequencing at approximately 20× coverage. Since the aim was to identify fixed genetic differences between two extremely divergent genotypes rather than to estimate population parameters, a single sample per genotype is sufficient for this purpose. Nevertheless, with a single biological replicate per cultivar, variants that are truly cultivar-characteristic cannot be formally distinguished from individual-specific private variants. The differences reported in this study should therefore be interpreted as characterizing the two specific accessions examined, and their generalization to broader cold-tolerant/cold-sensitive lily germplasm requires validation across additional cultivars and individuals.

All samples were freeze-dried, ground in liquid nitrogen, and weighed at 50 mg. Total DNA was extracted using the Plant Genomic DNA Extraction Kit from Tiangen Biotech (Beijing) Co., Ltd. (Beijing, China). Absorbance values (260/280 nm and 260/230 nm) were measured for each sample using a NanoDrop ND–2000 (Thermo Fisher Scientific, Waltham, MA, USA) to assess sample quality and concentration. DNA integrity was assessed via 1% agarose gel electrophoresis. DNA samples meeting strict quality standards were selected and stored at −20 °C for subsequent use. The qualified samples were then submitted to Beijing Biomarker Technologies Co., Ltd. (Beijing, China) for library construction and subsequent DNA sequencing.

### 2.3. Data Quality Control and Comparison

Raw sequencing data quality was assessed using FastQC (v0.11.9). Raw reads were filtered using fastp (v0.20.0) according to the standard whole-genome resequencing quality-control workflow: reads containing adapter sequences were removed (adapter trimming); reads with more than 10% N bases were removed; reads composed entirely of A bases were removed; and low-quality reads, in which bases with a quality score ≤ Q20 accounted for more than 50% of the read length, were removed (quality threshold). Quality trimming was performed using a sliding-window approach, and reads that were suboptimal in length after trimming were discarded (minimum read length), yielding high-quality clean data. The clean reads were aligned to the reference genomes of *L. davidii* var. *unicolor*, *L. sargentiae*, and *L. regale* using BWA-MEM2 with default parameters. The alignment results were sorted and deduplicated using SAMtools (v1.10). Mappability, average sequencing depth, and coverage at 1×, 5×, 10×, and 20× were calculated for each variety. The three reference genomes were evaluated based on read mapping rate, average sequencing depth, and genome coverage at multiple depth thresholds (1×, 5×, 10×, and 20×); the genome showing the highest mapping rate, adequate depth (≥20×), and the best coverage was selected as the primary reference for all downstream analyses.

### 2.4. SSR Identification and Characterization

SSRs were identified directly from the whole-genome sequences of the three reference genomes (*L. davidii* var. *unicolor*, *L. sargentiae*, and *L. regale*) using MISA (MIcroSAtellite identification tool, v2.1), rather than from the resequencing data. The following criteria were used for selection: ≥10 repeats for single-nucleotide repeats, ≥6 repeats for dinucleotide repeats, ≥5 repeats for trinucleotide repeats, and ≥4 repeats for tetra- to hexanucleotide repeats. These thresholds follow criteria commonly used in microsatellite identification studies, balancing detection sensitivity with the exclusion of spurious short repeat motifs. The output included the repeat unit type, number of repeats, start position, and end position for each SSR. Subsequently, the number of different SSR types and the distribution of repeat counts across the three genomes were statistically analyzed, and cumulative frequency curves were calculated. Additionally, potential polymorphic SSR loci with differences in repeat counts between ND-6 and ‘Sorbonne’ were identified.

### 2.5. InDel Detection and Annotation

Variants were detected using the HaplotypeCaller module of GATK (v4.6.2.0) on the alignment results (BAM files) of ND-6 and ‘Sorbonne’ against the L. davidii var. unicolor reference genome, generating per-sample gVCF files. The gVCFs of the two cultivars were combined with CombineGVCFs and jointly genotyped with GenotypeGVCFs. InDels were then extracted using SelectVariants v4.6.2.0 (--select-type-to-include INDEL). Subsequently, strict filtering was performed using VariantFiltration with the following criteria: QD < 2.0, QUAL < 30.0, FS > 200.0, and SOR > 10.0 (cluster-size 3); variants flagged by these filters were excluded, yielding a high-confidence InDel set. Functional annotation of the InDels was performed using snpEff (v5.0) against the *L. davidii* var. *unicolor* reference genome. The annotations included the genomic regions where the variants were located and their effects on coding sequences. The number of InDels in each region, the ratio of insertions to deletions, and the number of frameshift InDels in the CDS regions were calculated for each variety. Variants in low-coverage regions were not explicitly excluded; however, GATK’s likelihood-based genotyping tends to emit missing genotypes (no-calls) rather than false-positive variants at low depth. Incomplete coverage therefore primarily affects the detection rate (potentially under-estimating InDel counts) rather than the authenticity of the calls. The proportions of the reference genome retaining ≥10× and ≥20× sequencing depth are reported in Section 3.1.

### 2.6. SSR Amplification and DNA Electrophoresis

Primers were designed using Primer Premier 5 software with an expected amplicon size of 200–500 bp. The primer length (18–27 bp), Tm (42–58 °C), and GC content (30–52%) of the 20 primer pairs are listed in Supplementary Table S3. PCR amplification was performed with a uniform annealing temperature of 58 °C using the rTaq PCR Kit (Takara, Kusatsu, Shiga, Japan), with each sample prepared in a 20 µL reaction mixture. The reaction mixture contained: 20 ng genomic DNA, 10× rTaq buffer, 0.2 mM dNTP mixture, 1 μL each of forward/reverse primers, and 0.5 μL rTaq DNA polymerase. The PCR program commenced with a 5-min pre-denaturation step at 95 °C, followed by 30 cycles of: 1-min denaturation at 95 °C, 1-min annealing at 58 °C, and 1-min extension at 72 °C. The cycle concluded with a 5-min final extension at 72 °C.

Subsequently, samples were prepared by mixing 10 µL of PCR product with 3 µL of bromophenol blue loading dye. PCR products were separated by electrophoresis on 2% agarose gels using the wide mini-sub cell GT horizontal electrophoresis system (1,704,469, Bio-Rad Laboratories, Hercules, CA, USA), run in 1× Tris-acetate-EDTA (TAE) buffer at 120 V for 20 min, and visualized after electrophoresis. SSR bands were scored in a presence/absence (dominant) manner, and only clear, reproducible main bands were counted; stutter (shadow) bands and weak bands were excluded from the analysis. The polymorphism rate was defined as the proportion of polymorphic bands among all scorable bands.

## 3. Results

### 3.1. Quality Assessment of Whole-Genome Resequencing Data and Selection of Reference Genomes

Through DNA resequencing, we obtained genomic DNA sequences from the cold-tolerant lily cultivar “ND-6” and the non-cold-tolerant lily cultivar “*Lilium* ‘Sorbonne’”. Following quality control and data screening procedures, we ultimately obtained sequencing data from both samples. The samples yielded 3,062,792,510 and 2,762,666,556 high-quality reads, respectively, along with approximately 917 Gb and 828 Gb of bases (Table 1), indicating substantial sequencing depth. Further analysis of sequencing quality metrics revealed exceptionally high Q20 values of 99.55% and 99.29%, and Q30 values of 98.09% and 97.46% for the two samples, respectively, indicating outstanding sequencing accuracy. Additionally, the average GC content was 35.2% and 34.4%, respectively, falling within the normal range and further enhancing the reliability of the sequencing results.

**Figure 1:**
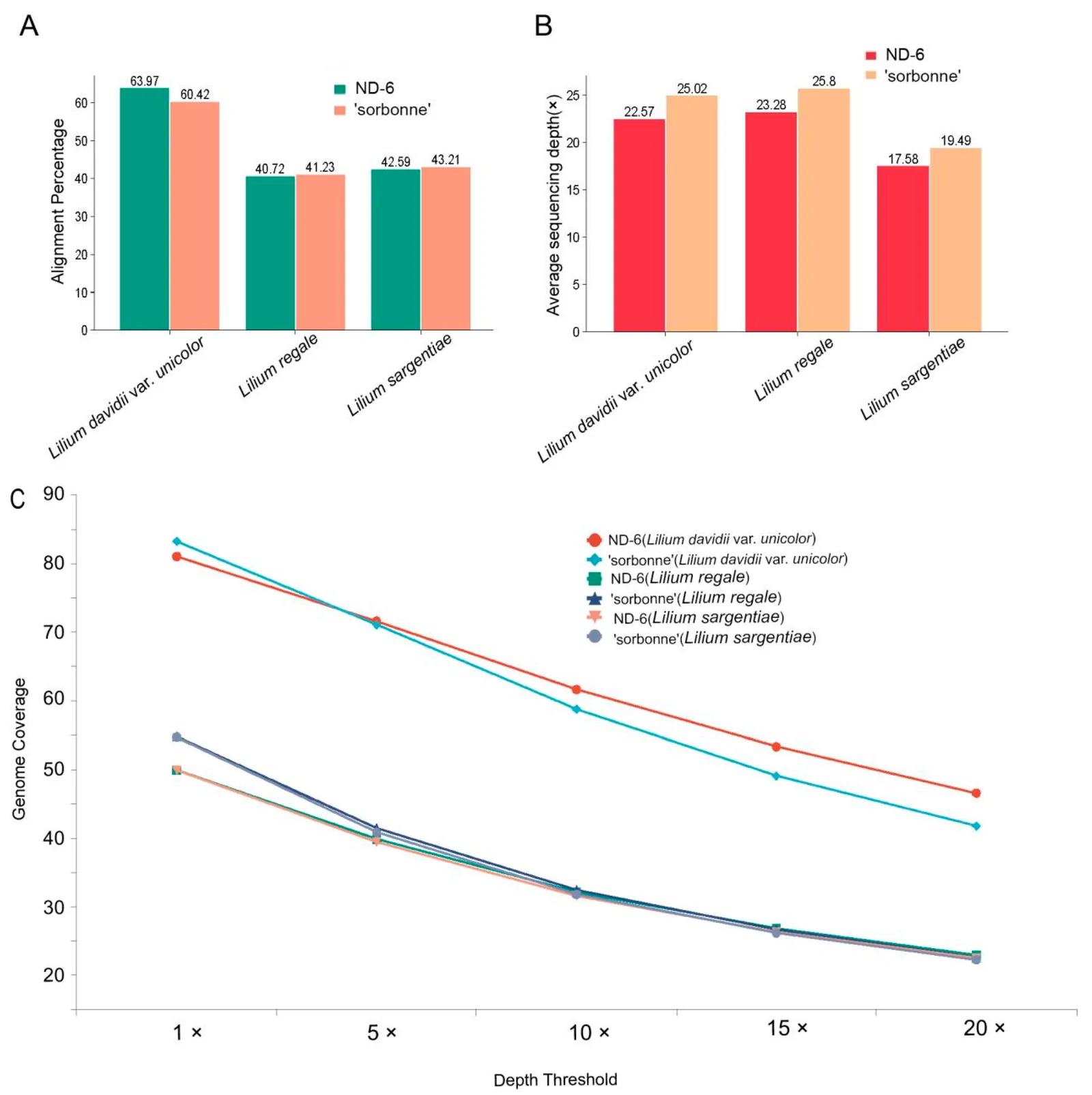
Quality assessment of whole-genome resequencing data and selection of reference genomes. (**A**) The bar chart shows the mapping rates (%) of sequencing reads from ND-6 and ‘Sorbonne’ to the three reference genomes. The x-axis represents the reference genomes, and the y-axis represents the mapping rate. (**B**) The bar chart shows the average sequencing depth (×) of the two varieties mapped to the three reference genomes. The x-axis represents the reference genome, and the y-axis represents the average sequencing depth. (**C**) A scatter plot with connecting lines shows the genome coverage curves for the two varieties against the three reference genomes. The x-axis represents depth thresholds (1×, 5×, 10×, 15×, 20×), and the y-axis represents the proportion of the genome covered at or above that depth (%).

To evaluate the suitability of three published lily genomes as reference genomes, were aligned to *L. davidii* var. *unicolor*, *L. regale*, and *L. sargentiae*. ND-6 achieved the highest mapping rate of 63.97% on *L. davidii* var. *unicolor*, followed by *L. sargentiae* at 42.59% and *L. regale* at 40.72%. ‘Sorbonne’ showed a similar trend, with mapping rates of 60.42%, 43.21%, and 41.23% for *L. davidii* var. *unicolor*, *L. sargentiae*, and *L. regale*, respectively (Figure 1A, Supplementary Table S1).

The average sequencing depths of ND-6 against the genomes of *L. davidii* var. *unicolor*, *L. regale*, and *L. sargentiae* were 22.57×, 23.28×, and 17.58×, respectively, while those of ‘Sorbonne’ were 25.02×, 25.80×, and 19.49×, respectively. Both varieties achieved the target depth of 20× for *L. davidii* var. *unicolor* and *L. regale*, while the depth was slightly lower for *L. sargentiae* (Figure 1B).

To gain a more intuitive understanding of the genome coverage of the two sequenced varieties when mapped to the three reference genomes, we plotted genome coverage curves at sequencing depths of 1×, 5×, 10×, 15×, and 20× (Figure 1C). The results showed that *L. davidii* var. *unicolor* exhibited the best genomic coverage. At a depth threshold of 1×, ND-6 covered 81.04% of the genome, while ‘Sorbonne’ covered 83.23%; at 10×, coverage dropped to 61.63% and 58.74%, respectively; even at the target depth of 20×, 46.49% (ND-6) and 41.74% (‘Sorbonne’) of the genome remained covered, indicating good sequencing uniformity and data quality sufficient to support subsequent SSR and InDel analyses. It should be noted, however, that about 38–42% and 54–58% of the genome fell below 10× and 20× depth, respectively; variants in these low-coverage regions may be under-detected.

Based on a comprehensive evaluation of alignment rate, sequencing depth, and coverage consistency, *L. davidii* var. *unicolor* was selected as the primary reference genome for all subsequent analyses (Supplementary Table S1). The flower morphology of the two cultivars examined in this study is shown in Supplementary Figure S2.

### 3.2. SSR Characteristics in Three Reference Genomes

**Figure 2:**
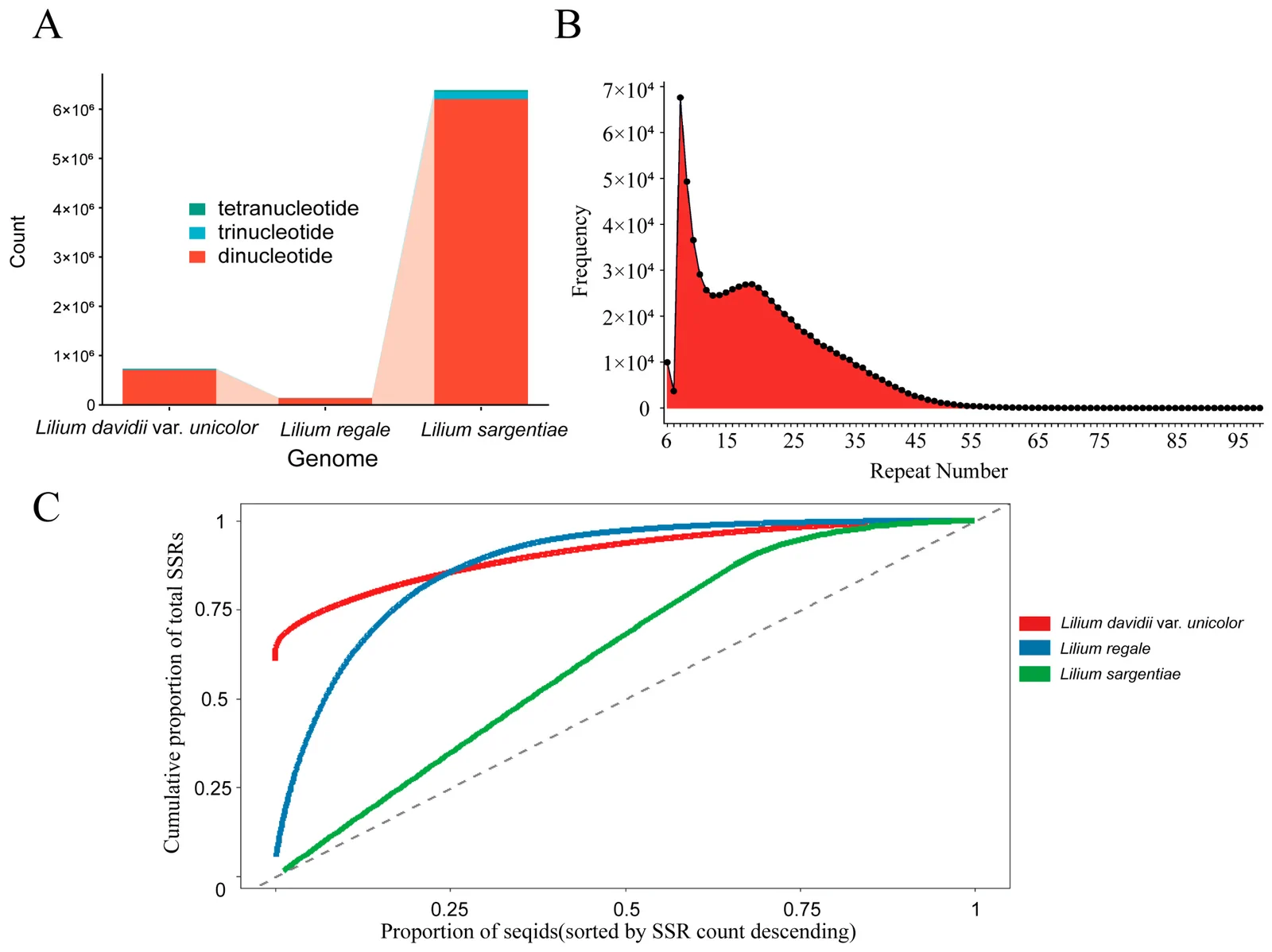
Genome-wide characterization of SSRs in three lily reference genomes. (**A**) Number of SSRs for each repeat type (di-, tri- and tetra-nucleotide) in *L. davidii* var. *unicolor*, *L. sargentiae* and *L. regale*. (**B**) Distribution of repeat numbers of all SSRs identified in *L. davidii* var. *unicolor*. (**C**) Cumulative proportion curves of SSRs for the three reference genomes. For each genome, seqids (contigs/scaffolds) were sorted in descending order of their SSR count. The x-axis represents the cumulative proportion of seqids, and the y-axis represents the cumulative proportion of total SSRs. The dashed diagonal line indicates an even distribution (i.e., SSR proportion equals seqid proportion). Curves that lie above the diagonal indicate that a small fraction of seqids contains a large fraction of SSRs.

To investigate the SSR characteristics of the lily genome, we identified SSRs in three published reference genomes (*L. davidii* var. *unicolor*, *L. sargentiae*, and *L. regale*). The results revealed significant variation in the total number of dinucleotide, trinucleotide, and tetranucleotide repeats analyzed across the three genomes (Figure 2A, Supplementary Table S2). In all three genomes, dinucleotide repeats were the most abundant, followed by trinucleotide repeats, with tetranucleotide repeats being the least abundant. Specifically, *L. davidii* var. *unicolor* contained 712,707 dinucleotide, 17,012 trinucleotide, and 3137 tetranucleotide SSRs; *L. sargentiae* contained 6,204,975, 154,663, and 25,758, respectively; and *L. regale* contained 137,256, 3697, and 530, respectively (Supplementary Table S2). We further analyzed the distribution of SSR repeat numbers using *L. davidii* var. *unicolor* as a representative. The results showed that SSRs with a frequency of 20,000 or higher had repeat numbers ranging from 8 to 24. The peak frequency occurred at 8 repeats, accounting for 9.2% of all SSRs. SSRs with more than 60 repeats were rare, with a frequency of less than 100, accounting for less than 0.1% of all SSRs (Figure 2B). To assess whether the distribution of SSRs across different seqids (contigs/scaffolds) is uniform, we plotted cumulative proportion curves and scatter plots (Supplementary Figure S1). For each genome, all seqids were sorted by the number of SSRs from highest to lowest, and curves were plotted with the cumulative proportion of seqids on the x-axis and the cumulative proportion of SSRs on the y-axis (Figure 2C). In all three genomes, the cumulative SSR proportion increased much more rapidly than the cumulative seqid proportion, and the curves all lay above the dotted diagonal line. The curve for *L. sargentiae* was closer to the dotted line than the other two, indicating that its SSR distribution was more uniform than that of the other two. In contrast, in the genomes of *L. davidii* var. *unicolor* and *L. regale*, the top 25% of seqids by number of SSRs contained more than 75% of the SSRs, indicating a highly uneven SSR distribution, with a small number of large seqids containing the majority of SSRs.

### 3.3. Genome-Wide Identification and Comparative Analysis of InDels in the Two Varieties

**Figure 3:**
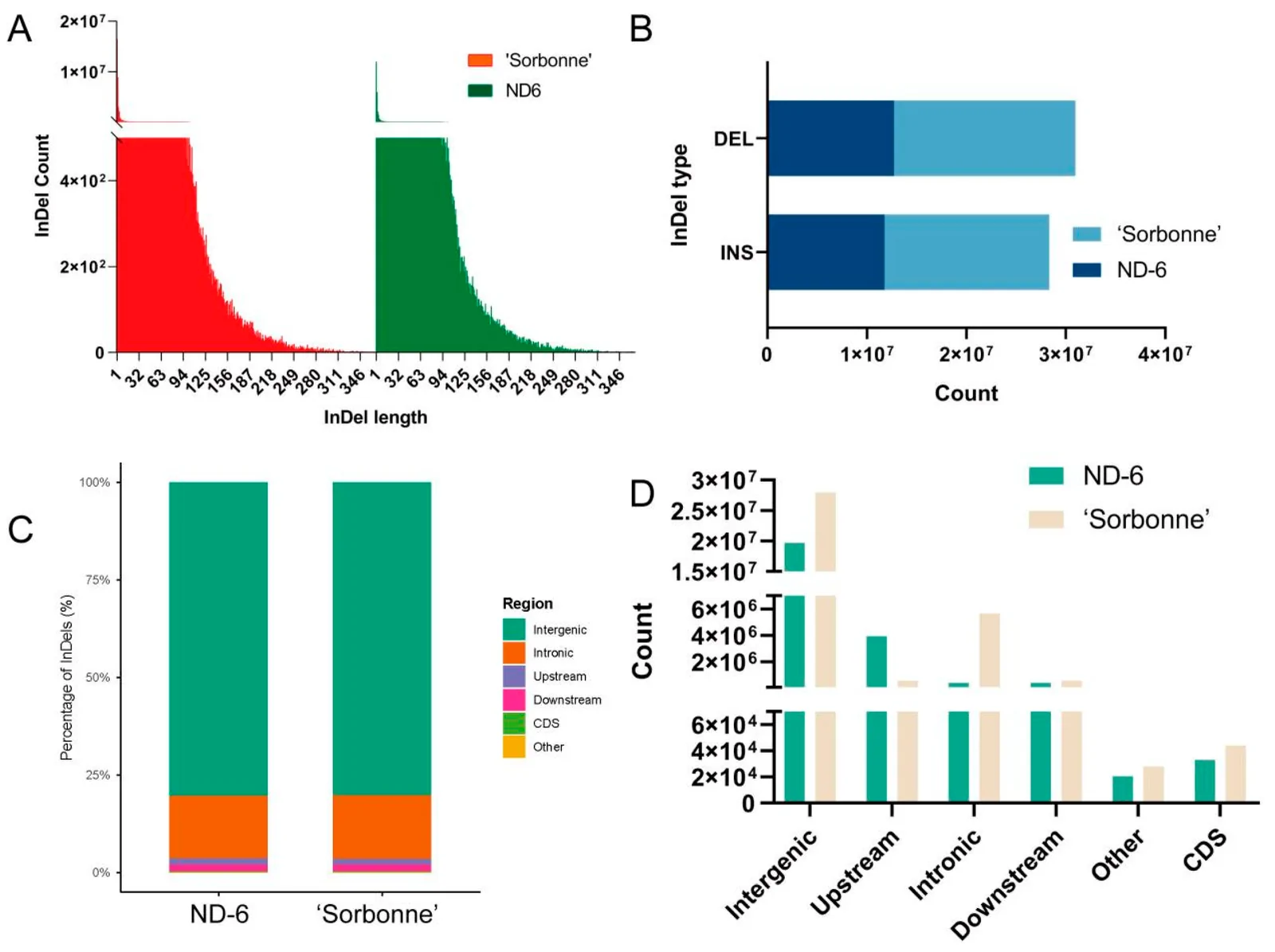
Genome-wide identification and comparative analysis of InDels in the two varieties. (**A**) Length distribution of InDels. The x-axis represents InDel length (bp), and the y-axis represents the number of InDels. (**B**) Ratio of insertions (INSs) to deletions (DELs). (**C**) Relative distribution of InDels across different genomic regions (percentage). (**D**) Number of InDels in each genomic region for the two varieties.

To investigate InDel variations between two lily cultivars with different cold hardiness, we aligned resequencing reads from ND-6 and ‘Sorbonne’ to the *L. davidii* var. *unicolor* reference genome, performed InDel detection using GATK, and conducted functional annotation with snpEff. In the joint genotyping call set, a total of 768,912,582 variants (including SNPs, InDels, and other types) were identified, of which 51,104,086 were InDels. After strict filtering (QD < 2.0, QUAL < 30.0, FS > 200.0, SOR > 10.0), 50,980,120 InDels (99.76%) passed the quality filters (Supplementary Table S4). A total of 34,812,909 InDels were identified in ‘Sorbonne’ and 24,497,857 InDels in ND-6. 1-bp InDels were the most abundant category in both varieties, with 16,445,728 in ‘Sorbonne’ and 11,891,364 in ND-6. This was followed by 2-bp InDels, with 8,825,909 in ‘Sorbonne’ and 5,878,438 in ND-6 (Figure 3A). The number decreased rapidly with increasing length, and InDels longer than 50 bp accounted for less than 0.1% of the total. In all InDels, deletions (DELs) consistently outnumbered insertions (INSs) in both varieties (Figure 3B). In ‘Sorbonne’, DELs accounted for 18,249,947 (52.4%), while INSs accounted for 16,562,962 (47.6%). In ND-6, DELs accounted for 12,720,536 (51.9%) and INSs for 11,777,321 (48.1%). Overall, the DEL/INS ratio was approximately 1.1:1, indicating a slight but consistent deletion bias in the lily genome. Functional annotation using snpEff revealed that the majority of InDels were located in intergenic regions, with 27,928,909 in ‘Sorbonne’ and 19,702,014 in ND-6; ‘Sorbonne’ accounted for 80.23% and ND-6 for 80.42% (Figure 3C). InDels within introns constitute the second most abundant category, accounting for 16.28% in ‘Sorbonne’ and 16.03% in ND-6. Furthermore, the number of InDels located in the coding region (CDS) was very low. Specifically, ‘Sorbonne’ had 44,029 (0.126%), and ND-6 had 32,975 (0.135%). InDels in upstream, downstream, and other annotated regions combined accounted for less than 5% of the total (Figure 3D). Notably, the number of upstream InDels was higher in ND-6 than in ‘Sorbonne’, but the opposite was true for other regions. These InDels may affect the expression of nearby genes by altering promoters or transposons. The CDS, upstream, and intronic InDel counts should be interpreted with this coverage background in mind (Section 3.1); low-coverage regions may lead to under-detection rather than false-positive calls.

### 3.4. Screening and Validation of Polymorphic SSR Markers

**Figure 4:**
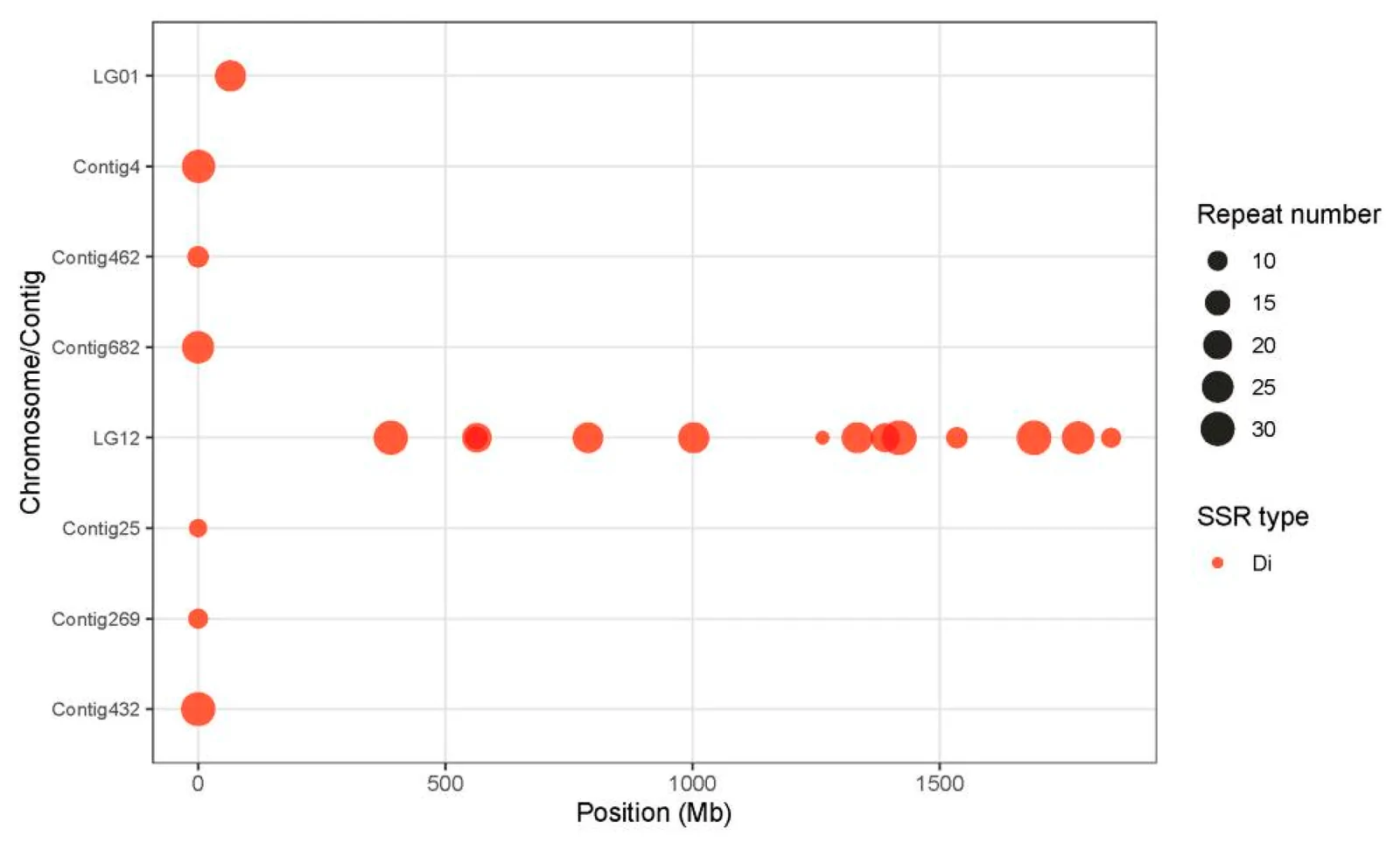
Selection of candidate SSR markers and validation of polymorphisms. Distribution of 20 candidate SSR loci on the reference genome. Each dot represents an SSR and is plotted based on its physical location (Mb) on the corresponding contig or chromosome (seqid). The size of the dots is proportional to the number of repeats. All selected markers are dinucleotide repeats (Di).

To obtain SSR markers suitable for experimental validation, we first screened the *L. davidii* var. *unicolor* reference genome for SSRs with a repeat number ≥ 6. From this set, we selected 20 loci distributed across different contigs and chromosomes, spanning a wide range of repeat numbers (8–31) and genomic positions (from ~44 kb on Contig 462 to ~1760 Mb on LG12), to ensure genome-wide representation and avoid excessive clustering within short genomic regions (Figure 4). Among the 20 candidate loci, 6 were located on contigs, 13 on chromosome LG12, and 1 on chromosome LG01, with position coordinates ranging from approximately 44 kb (Contig 462) to approximately 1760 Mb (LG12). All selected SSRs are dinucleotide repeats, with repeat numbers ranging from 8 to 31 and an average of 20.5. This selection covers a wide range of repeat lengths and genomic backgrounds, helping to ensure that the designed primers are suitable for PCR polymorphism screening between ND-6 and ‘Sorbonne’.

### 3.5. Validation and Analysis of SSR Molecular Markers

**Figure 5:**
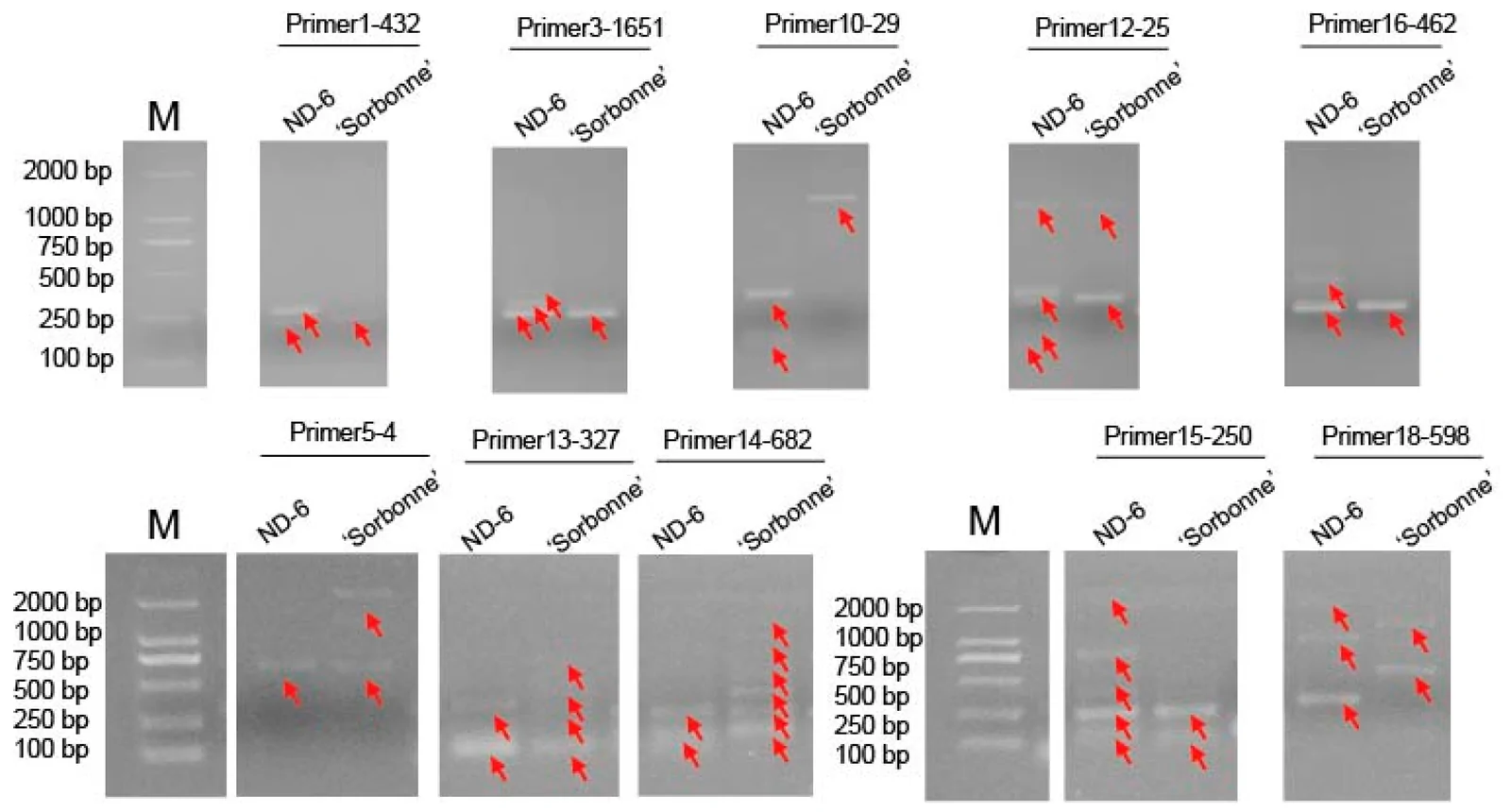
Validation and analysis of SSR molecular markers. The products amplified by the SSR primers were analyzed and identified using 2% agarose gel electrophoresis. M: DNA marker. The red arrows indicate the positions of the bands.

To screen for primers suitable for the molecular identification of cold-tolerant lily germplasm, we designed 20 pairs of SSR primers. From the 20 candidate primer pairs, 10 pairs that produced clear bands and showed good reproducibility were selected for polymorphism analysis (Figure 5, Supplementary Table S3). The repeat motifs of these 10 primer pairs were all dinucleotides (Di). PCR amplification was performed at a uniform annealing temperature of 58 °C. The 10 pairs of primers amplified a total of 46 distinct bands, of which 38 were polymorphic, resulting in a polymorphism rate of 82.6% (a band-based rate defined as the proportion of polymorphic bands among all scorable, clear, reproducible main bands). Each primer amplified 3–9 bands, with an average of 4.6 bands. Primer 14-682 amplified the most bands (9), while primers 1-432, 16-462, and 5-4 amplified the fewest, with 3 bands each.

The results indicate that the 10 pairs of SSR primers identified exhibit high polymorphism between ND-6 and ‘Sorbonne’ and can effectively distinguish between the two cultivars examined in this study, thereby providing a molecular foundation for subsequent genetic diversity analysis and variety identification. Their potential utility in marker-assisted selection, however, requires validation through association or linkage analyses.

## 4. Discussion

This study conducted whole-genome DNA resequencing on two lily varieties with significantly different cold tolerance, the cold-tolerant variety ND-6 and the cold-sensitive variety ‘Sorbonne’. Using the published chromosome-level reference genome of *L. davidii* var. *unicolor*, we systematically analyzed their SSR and InDel variations.

Through comparative analysis of the data, we demonstrated the validity of selecting this reference genome. Compared with the genomes of two other published species, *L. sargentiae* and *L. regale*, the resequencing data from these two lily varieties showed the highest read alignment rates against *L. davidii* var. *unicolor*, at 63.97% and 60.42%, respectively. Furthermore, the sequencing depth was sufficient, exceeding 22×, and the genome coverage was optimal.

It is worth noting that even under the stringent depth threshold of 20×, nearly half of the *L. davidii* var. *unicolor* genome was effectively covered, indicating that the resequencing data is sufficiently homogeneous to support reliable variant detection. This step is crucial because the extremely high heterozygosity and massive size (30–48 Gb) of the lily genome have long hindered the systematic exploration of genome-wide polymorphisms [3,5]. The recently published lily genome thus provides a solid foundation for the comprehensive development of markers for plants in this genus [4,5]. It should be noted that the lily reference genome (36.68 Gb) is more than ten times the size of the human genome, with extremely high repeat content and heterozygosity; accordingly, the mapping rate (~60–64%) and coverage metrics reported here are lower than those typical of conventional plant resequencing studies and should be interpreted in light of this genomic scale. Because GATK-based genotyping emits missing calls rather than false positives at low depth, the main effect of incomplete coverage is under-detection rather than inflation of the InDel catalog.

Analysis of SSRs in the three reference genomes revealed that dinucleotide repeats were by far the most abundant, followed by trinucleotide and tetranucleotide repeats, a pattern consistent with that observed in other plant species [10]. However, there were significant differences in the total number of SSRs among the genomes; *L. sargentiae* contained over six million dinucleotide SSRs, nearly nine times that of *L. davidii* var. *unicolor*. This difference likely stems from variations in genome assembly continuity, genome size, and the content of repetitive sequences.

The SSR cumulative proportion curves further indicate that the distribution of SSRs across sequence units is highly uneven. In *L. davidii* var. *unicolor* and *L. regale*, the top 25% of sequences in terms of SSR abundance account for more than 75% of the SSRs across the entire genome. This tendency toward clustering is similar to findings in other large plant genomes. In these genomes, microsatellites tend to accumulate in gene-rich regions or regions enriched with repetitive sequences [19]. This distribution pattern suggests that their physical locations should be fully considered when genotyping with SSR markers to avoid biases resulting from locus clustering.

The large differences in total SSR numbers among the three genomes (e.g., *L. sargentiae* contained >6,000,000 dinucleotide SSRs, approximately nine times that of *L. davidii* var. *unicolor*) most plausibly reflect differences in assembly quality and scaffold fragmentation as well as repeat content, rather than purely biological variation. Because the SSR detection method (MISA) and parameters were identical across the three genomes, methodological differences can be excluded. Assembly continuity and repeat content should therefore be considered when comparing SSR numbers across genomes.

One of the key contributions of this study is the identification and comparative analysis of genome-wide InDels between two lily varieties. A total of 34,812,909 and 24,497,857 InDels were detected in ‘Sorbonne’ and ND-6, respectively. The length distribution of InDels showed a sharp decline with increasing length, with 1-bp InDels being the most abundant; this pattern is consistent with reports in rice [14], cucumber [16], and rapeseed [18]. Short InDels accounted for the majority of the total, reflecting the tendency of DNA polymerase slippage and repair mechanisms to generate small-fragment insertions or deletions [9]. The overall ratio of deletions to insertions in both varieties was approximately 1.1:1, indicating a slight preference for deletions. This trend is consistent with observations in other plants, such as barley [15] and Chinese cabbage [17,20]. Functional annotation revealed that over 80% of InDels are located in intergenic regions, with only about 0.13% situated within coding sequences (CDSs). This distribution suggests that the direct impact of most InDel variants on protein-coding sequences is likely limited. However, the substantial number of InDels located in upstream regions and introns may still regulate gene expression by affecting regulatory elements or splicing processes, warranting further investigation. It is worth noting that the total number of InDels differed substantially between the two cultivars (34,812,909 in ‘Sorbonne’ versus 24,497,857 in ND-6, a ~42% difference relative to the same reference genome). Given the comparable sequencing depth and base quality between the two samples (Table 1), this asymmetry is unlikely to arise primarily from technical factors. A more plausible explanation is the difference in genetic distance to the reference: ‘Sorbonne’ is an Oriental hybrid whose ancestry traces to Japanese wild lily species (e.g., *L. speciosum* and *L. auratum*) that are phylogenetically more distant from *L. davidii* var. *unicolor*, whereas ND-6 is a locally bred hybrid that is comparatively more similar to the reference lineage (consistent with its slightly higher mapping rate to *L. davidii* var. *unicolor*; Figure 1A). The greater sequence divergence of ‘Sorbonne’ from the reference, within comparably covered genomic regions, therefore most plausibly explains its higher InDel count. Because a larger fraction of reads from the more divergent cultivar remain unmapped, the true biological divergence may be even more pronounced than observed. This interpretation remains preliminary and awaits validation with whole-genome data from additional lily germplasm.

Through experimental validation of 10 pairs of selected SSR primers, we obtained a high polymorphism rate of 82.6%, with an average of 4.6 bands amplified per primer pair. This level of polymorphism is comparable to that reported for genomic SSR markers in crops such as cucumber [21] and rapeseed [22]. Since all validated markers are dinucleotide repeats and are distributed across different contigs and chromosomes, they can be used as a marker panel to effectively distinguish the cold-tolerant cultivar ND-6 from the cold-sensitive cultivar ‘Sorbonne’ examined in this study. Previously, SSR development in lilies has primarily relied on EST or transcriptomic data [11,23]. The genomic SSR markers developed in this study offer advantages at the DNA level, particularly in identifying variations in non-coding regions that may be associated with differences in cold tolerance regulation. Both cultivars are diploid or expected to be diploid: ‘Sorbonne’ has been described as a diploid (2n = 2x = 24) Oriental hybrid cultivar [24], and cultivars of the Oriental, Asiatic, and Longiflorum hybrid groups are predominantly diploid [25]; ND-6 is a newly bred hybrid cultivar whose male parent is an Oriental hybrid, and formal ploidy determination of ND-6 is pending. Regarding the relatively high number of bands amplified per primer pair (3–9, average 4.6), because both cultivars are (expected to be) diploid, the band multiplicity is most likely attributable to multi-locus amplification of paralogous sites in the highly repetitive, transposon-rich lily genome [5], with PCR stutter of dinucleotide repeats contributing additional shadow bands. Such multi-locus amplification is common for genomic SSR markers in complex genomes. In future work, these markers could be converted to single-locus systems using single-copy InDel markers or high-throughput genotyping approaches (e.g., genotyping-by-sequencing).

Because no association or linkage analysis was performed in this study, the association between these markers and cold-tolerance loci or phenotypes has not been established; any application in marker-assisted selection therefore requires further validation through association or linkage analyses in a broader germplasm panel or segregating population.

This study also has some limitations. First, whole-genome resequencing was performed on a single individual per cultivar (ND-6 and ‘Sorbonne’). The differences identified cannot formally distinguish cultivar-characteristic variants from individual-specific private variants, and the markers are therefore best regarded as distinguishing these two specific accessions; their utility for broader cold-tolerant/cold-sensitive germplasm discrimination requires validation across additional cultivars and individuals. Second, although we identified SSR molecular markers, we have not yet established an association between these markers and the cold tolerance phenotype. Third, no Sanger (or other independent) validation of the called InDels was performed; given the highly repetitive, heterozygous mega-genome and short-read variant calling, a subset of InDels, especially in intergenic/repetitive regions, may include alignment artifacts, and independent sequencing validation of representative InDel loci is planned for future work. Fourth, this study focused only on two- to four-nucleotide SSR markers, and other types of repeats were not analyzed. Fifth, validation of these markers across a broader panel of cold-tolerant/cold-sensitive lily cultivars and accessions is required to assess their robustness, discriminatory power, and practical utility in large-scale germplasm screening, and is planned in our ongoing breeding program. In this study, SSR genotyping was performed using 2% agarose gel electrophoresis, which offers lower resolution than polyacrylamide gel electrophoresis (PAGE); to further improve genotyping resolution, PAGE-based validation of representative markers is planned in future work. In addition, the polymorphic SSR bands were not confirmed by band-excision sequencing; Sanger sequencing of representative polymorphic bands is planned for future work.

In summary, this study conducted a genome-wide characterization of InDels in lilies and experimentally validated polymorphic SSR markers that can be used to distinguish the cold-tolerant cultivar ND-6 from the cold-sensitive cultivar ‘Sorbonne’. The genomic resources and markers generated in this study not only enrich research on lily genetics but also provide a foundation for future genetic mapping, diversity analysis, and the potential application of these markers in marker-assisted selection for cold tolerance, pending association or linkage analyses.

## 5. Conclusions

In this study, whole-genome resequencing was performed on the cold-tolerant variety ND-6 and the cold-sensitive variety ‘Sorbonne’, and a systematic analysis of genome-wide SSR and InDel variations was conducted. Among the three candidate reference genomes, the *L. davidii* var. *unicolor* genome was selected as the most suitable reference based on its optimal alignment rate, sufficient sequencing depth, and best genomic coverage. A comparative InDel analysis identified 34.81 million and 24.50 million InDels in ‘Sorbonne’ and ND-6, respectively. Short InDels (1–2 bp) predominated, with a slight but consistent preference for deletions. More than 80% of the InDels were located in intergenic regions, indicating that their direct impact on coding sequences is limited. Experimental validation of 10 polymorphic SSR markers revealed a polymorphism rate of 82.6%, demonstrating their effectiveness in distinguishing between the two cultivars examined in this study. The genomic resources and molecular markers generated in this study provide an important foundation for lily germplasm identification and genetic diversity assessment, and for future evaluation of their utility in marker-assisted selection. Future association analyses linking phenotypic data from large-scale populations with these variants will be crucial for advancing their application in cold-tolerance breeding.

## Figures and Tables

**Table 1:** Statistics on sequencing data.

Sample	Obtained Reads	Obtained Base (Gb)	Q20 (%)	Q30 (%)	GC (%)
ND-6	3,062,792,510	917.6677598	99.55	98.09	35.2
*Lilium* ‘Sorbonne’	2,762,666,556	827.9435666	99.29	97.46	34.4

Note: Q20 (%) and Q30 (%) represent the percentage of bases in the original sequence with quality values greater than 20 and 30 (error rates less than 0.1% and 0.1%, respectively). The clean reads and clean bases reported in Table 1 refer to the data after QC; dividing the clean bases by the reference genome size (36.68 Gb) yields the nominal average depth (~20×). The actual average depth and genome coverage after read mapping (≥1×, ≥5×, ≥10×, ≥20×) are reported in Section 3.1.

## Data Availability

The raw sequence data reported in this paper have been deposited in the Genome Sequence Archive (Genomics, Proteomics & Bioinformatics 2025) in the National Genomics Data Center (Nucleic Acids Res 2025), China National Center for Bioinformation/Beijing Institute of Genomics, Chinese Academy of Sciences (GSA: CRA047103), which are publicly accessible at https://ngdc.cncb.ac.cn/gsa (accessed on 20 July 2026).

## Supplementary Materials

The following supporting information can be downloaded at: https://www.mdpi.com/article/10.3390/biology15171449/s1, Figure S1: Distribution of SSRs across seqids in three lily reference genomes; Figure S2: Phenotype photographs of the two lily cultivars examined in this study. Table S1: Quality Assessment of Whole-Genome Resequencing Data and Selection of Reference Genomes; Table S2: Number of SSR Types; Table S3: 20 pairs of SSR primer sequences; Table S4: InDel variant detection and filtering statistics for ND-6 and ‘Sorbonne’ against the *L. davidii* var. *unicolor* reference genome.
