# Supplementary material for: Genome-Wide Identification of SSR and InDel Markers and Experimental Validation of SSR Markers for Distinguishing Cold-Tolerant and Cold-Sensitive Lily Cultivars
Source: Biology (Basel). 2026 Aug 24;15(17):1449. doi: 10.3390/biology15171449 (PMC13564514; doi:10.3390/biology15171449)
Supplement: Supplementary file 1 [file biology-15-01449-s001.zip › biology-4485273-supplementary.pdf]

Article

Simple Sequence Repeats (SSRs) and Cold Resistance Related Gene Analysis of Cold Resistant and Non Cold Resistant Lilies

Yanan Lu[1,] , Mingliang He[3], Lei Wang[3], Meng Zhao[1], Qianting Liu[1], Hongmei Bai[1], Yan Liu[1], Jing Gao[1], Li Zhang[1], Zhaoxin Wu[1], Zhi Liu[2,]*, Yanan Wang[1,]*

[1] Inner Mongolia Academy of Agricultural and Animal Husbandry Science, Hohhot 010031, China
[2] Guangdong Provincial People's Hospital, Guangdong Academy of Medical Sciences, Guangzhou 510080, China
[3] Institute of Microbiology, Xinjiang Academy of Agricultural Sciences, Urumqi 830091, Xinjiang, China
[*] Correspondence: ynwangnefu@163.com (Y.W.); liuzhi1@gdsrmyy.wecom.work (Z.L.)

Supplementary Data:

Supplementary Figure S1:

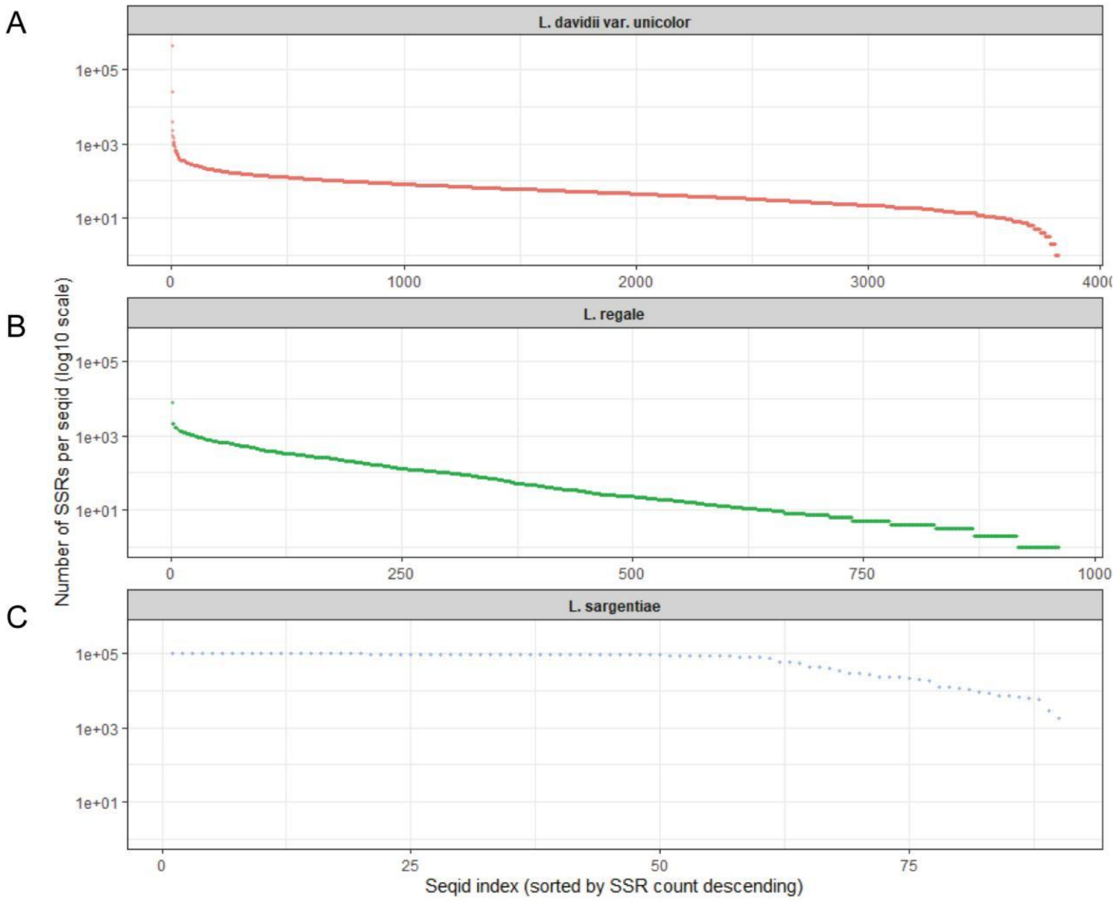

Supplementary Figure S1: Distribution of SSRs across seqids in three lily reference genomes. Scatter plots for (a) *Lilium davidii* var. *unicolor*, (b) *Lilium sargentiae*, and (c) *Lilium regale*. In

each panel, each dot represents a single seqid (contig/scaffold). The x-axis shows the seqid index after sorting all seqids in descending order of their SSR count (i.e., the leftmost seqid contains the most SSRs). The y-axis indicates the number of SSRs per seqid on a $\log_{10}$ scale.

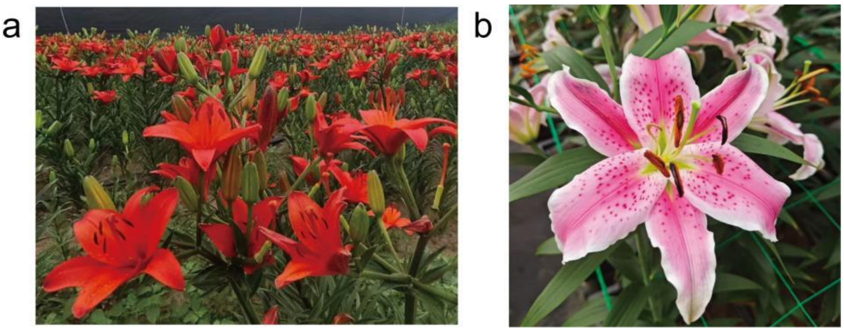

Supplementary Figure S2. Phenotype photographs of the two lily cultivars examined in this study. (a) The cold-tolerant cultivar 'ND-6' and (b) the cold-sensitive Oriental hybrid 'Sorbonne', showing whole-plant habit and flower/inflorescence morphology.

Supplementary Table S1. Quality Assessment of Whole-Genome Resequencing Data and Selection of Reference Genomes

Sequencing Varieties	Reference Genome	Total number of reads	Number of mapped reads	Alignment Rate (%)	Average Depth (×)	1× Depth Threshold (%)	10× Depth Threshold (%)
ND-6	*Lilium davidii* var. *unicolor*	3062792510	20626300979	63.9693%	22.57210787	81.04%	61.63%
ND-6	*Lilium sargentiae*	3062792510	16557301946	42.5929%	17.58133451	49.88%	31.52%
ND-6	*Lilium regale*	3062792510	13126871916	40.7181%	23.27698396	49.91%	32.09%
Sorbonne’	*Lilium davidii* var. *unicolor*	2762666556	17639480956	60.4205%	25.0182458	83.23%	58.74%
Sorbonne’	*Lilium sargentiae*	2762666556	15190313668	43.2147%	19.48662265	54.65%	31.77%
Sorbonne’	*Lilium regale*	2762666556	12016366421	41.227%	25.79950927	54.76%	32.40%

Supplementary Table S2. Number of SSR Types

genome	ssr_type	count
Lilium davidii var. unicolor	trinucleotide	17012
Lilium davidii var. unicolor	dinucleotide	712707
Lilium davidii var. unicolor	tetranucleotide	3137
Lilium regale	trinucleotide	3697
Lilium regale	dinucleotide	137256
Lilium regale	tetranucleotide	530
Lilium sargentiae	trinucleotide	154663
Lilium sargentiae	dinucleotide	6204975
Lilium sargentiae	tetranucleotide	25758

Supplementary Table S3. 20 pairs of SSR primer sequences

Primer name	Primer sequence	Base number	Tm (℃)	GC (%)
P1-432-F	CCACCCAACTTATCCATCATATGTACC	27	58	44
P1-432-R	CTTCACACTCAAGTGATGTGTTACATG	27	57	41
P2-1423-F	CCTGAGTACTTCAGATCAGAC	21	52	47
P2-1423-R	GAATCCGTAAAGCCTTGTAGC	21	52	48
P3-1651-F	CCACCTTCACTAGTCGATTATC	22	54	45
P3-1651-R	GTGGATACCCTTATCACCTCTTG	23	57	48
P4-1582-F	gtaacatatcttcttcccatcg	22	51	41
P4-1582-R	taaaggaaatattagggttc	20	43	30
P5-4-F	GTTACCCAAGACTTGTGG	18	48	50
P5-4-R	GATAGTTAAGTCACAATACC	20	46	35
P6-291-F	GTAGAGTAAGTAATGATAAGG	21	46	33
P6-291-R	GAAACAATCCTCTCTCTCACTC	22	54	45
P7-1997-F	GTAGAATGAATGCACATGATGAG	23	51	39
P7-1997-R	CATTAGAACTCAACTTATCAAC	22	47	32
P8-126-F	CACATCATATTAAATAATCTCCCAC	25	58	48
P8-126-R	CTTCTCTATGGGTATCCAATG	21	51	32
P9-677-F	GAAGTCATATGTTGTTGTGGTC	22	50	43
P9-677-R	CCAAAATGATGCCACTTAACATGAC	25	54	40
P10-29-F	CTACAGTCAACCAATAATGACATG	24	52	37
P10-29-R	CACCATCTTATTCTCCACTAGTC	23	53	43
P11-269-F	GTGTGTGTATGTTTAGATGTG	21	48	38
P11-269-R	CACATGCATTTACATACACACAC	23	50	35
P12-25-F	GATGACTTGATATGATCAATTGG	23	50	35
P12-25-R	GGTTGGTTAAGAATGGTGTTG	21	50	43
P13-327-F	GGTACCAGAGTGTTAGATATG	21	50	44
P13-327-R	TGACCACCATATGTCAATCGAC	22	54	45

P14-682-F	CCATTATCGTCGATATCTAG	20	48	40
P14-682-R	TACCCTCTTGAGTTGTGTCC	20	52	50
P15-250-F	ATCTTCCTACTTCACCCCAGTTC	23	55	48
P15-250-R	GCAAGAGATATTGATAGCAG	20	48	40
P16-462-F	GCTACTCAACAGTGAGTGTC	20	52	50
P16-462-R	CATAAGTCCGTTATGTCTCC	20	50	45
P17-2-F	GTCACAACCCATTGAGTTGG	20	58	42
P17-2-R	GATTCCATATCTGTTTGACTCG	22	51	41
P18-598-F	GTGGGATACATGTTATCTAT	20	42	35
P18-598-R	GAACTAGTATTTGAGGTTTGG	21	48	38
P19-151-F	CACCGCCTGGTAAACTAAGTC	21	54	52
P19-151-R	GTTGATATGTGTACTGAGTGG	21	50	44
P20-3239-F	GAGCTAGAGATCTTTCATACTC	22	51	41
P20-3239-R	ATCGTGTAGATATCCTGCTAGC	22	53	45

Supplementary Table S4. InDel variant detection and filtering statistics for ND-6 and 'Sorbonne' against the *L. davidii* var. *unicolor* reference genome.

Stage / Item	Count
Total raw variants (GenotypeGVCFs) (including SNPs, InDels, and other types)	768,912,582
Raw InDels (SelectVariants)	51,104,086
PASS InDels after VariantFiltration	50,980,120
Retention rate (%)	99.76
InDels flagged by filters (total)	123,966
QD < 2.0	123,929
FS > 200.0	5
SOR > 10.0	35
QUAL < 30.0	0
